# Machine learning in diabetic-foot osteomyelitis: current applications, challenges, and future directions

**DOI:** 10.5194/jbji-11-623-2026

**Published:** 2026-10-13

**Authors:** Farzad Pourghazi, Seyed Mohammad Amin Alavi, Francesco Petri, Takahiro Matsuo, Sergio Alvarez Mulett, Fabio Borgonovo, Andrea Gori, Michael E. Torchia, Aaron J. Tande, Elie F. Berbari

**Affiliations:** 1 Division of Public Health, Infectious Diseases and Occupational Medicine, Department of Medicine, Mayo Clinic College of Medicine and Science, Mayo Clinic, Rochester, MN, USA; 2 Faculty of Medicine, Ahvaz Jundishapur University of Medical Sciences, 6135715753 Ahvaz, Iran; 3 Department of Infectious Diseases, Luigi Sacco University Hospital, Regional Center for Infectious Diseases (CEREMI), 20157 Milan, Italy; 4 Department of Infectious Diseases, Infection Control, and Employee Health, The University of Texas MD Anderson Cancer Center, Houston, TX, USA; 5 Department of Biomedical and Clinical Sciences, University of Milan, 20157 Milan, Italy; 6 Centre for Multidisciplinary Research in Health Science (MACH), University of Milan, 20157 Milan, Italy; 7 Department of Orthopedic Surgery, Mayo Clinic, Rochester, MN, USA

## Abstract

**Background**: Diabetic-foot osteomyelitis (DFO) is a severe complication of diabetic-foot ulcers associated with delayed healing, amputation, and increased mortality. Machine learning (ML) has increasingly been explored for diagnosis and clinical decision-making in diabetic-foot care; however, the current evidence specific to DFO has not been systematically synthesized. **Methods**: We performed systematic searches of MEDLINE, Embase, Scopus, and Web of Science for studies published between January 2000 and May 2026. Studies that developed or evaluated ML models for DFO diagnosis, prediction, or outcomes were included. Data extraction was performed using the CHARMS framework, reporting followed PRISMA 2020 and TRIPOD-SRMA recommendations, and methodological quality was evaluated using PROBAST + AI. Due to considerable heterogeneity, narrative synthesis was performed. **Results**: A total of 10 studies published from 2022 to 2026 met the inclusion criteria. Five studies developed or evaluated ML models with a direct DFO diagnostic endpoint, one additional study evaluated model explainability, and four evaluated complementary DFO-relevant diabetic-foot applications. Reported discrimination ranged from 0.879 to 0.998, but comparisons were limited by differences in clinical endpoints, reference standards, input modalities and validation strategies. Only one direct DFO model was externally validated, and no study developed a multimodal model combining clinical and imaging data. **Conclusion**: Current evidence is limited by heterogeneous methodologies, limited external validation, and inconsistent reporting despite promising discrimination reported by several models.

Future studies should evaluate whether multimodal models integrating clinical and imaging data provide incremental value over unimodal approaches, with external validation, calibration, and clinical utility assessment before clinical implementation.

## 1 Introduction

Diabetic-foot ulcers (DFUs) are common and disabling complications of diabetes. Globally, an estimated 529 million adults had diabetes in 2021 (Ong et al., 2023), and 19 %–34 % will develop a DFU during their lifetime (McDermott et al., 2023). More than half of DFUs become infected, and approximately 20 % of moderate-to-severe diabetic-foot infections result in amputation (Armstrong et al., 2023). DFU is also associated with longer lengths of stay, greater emergency department utilization, and 2.5-fold-higher 5-year mortality than diabetes without DFU (Armstrong et al., 2023). The American College of Radiology reports that 20 % of patients with DFU will develop osteomyelitis (Baker et al., 2025).

Beyond amputation risk, diabetic-foot osteomyelitis (DFO) imposes substantial clinical and economic burdens, often requiring prolonged antibiotics, repeated testing and procedures, hospitalization, and multidisciplinary care (Giurato et al., 2017). The 2023 International Working Group on the Diabetic Foot/Infectious Diseases Society of America (IWGDF/IDSA) guideline recommends considering surgical resection of infected bone combined with systemic antibiotics in selected patients with DFO (Senneville et al., 2023), highlighting the importance of accurate diagnosis for treatment and limb preservation decisions.

Diagnosing DFO is challenging as no single test is accurate enough and because its findings overlap with other conditions, including Charcot neuroarthropathy, trauma, and soft-tissue infection (Senneville et al., 2023).

The IWGDF/IDSA guideline recommends initial studies including clinical assessment with probe-to-bone testing, plain radiography, and inflammatory biomarkers. Magnetic resonance imaging (MRI) is indicated if the diagnosis is uncertain and bone biopsy can be considered for microbiological or histopathological confirmation (Senneville et al., 2023).

Plain radiography has limited sensitivity (61.9 %) but moderate specificity (78.3 %) for DFO (Llewellyn et al., 2020), whereas serial imaging may improve sensitivity but reduce specificity (Harrison et al., 2026). MRI has higher diagnostic accuracy, with a reported sensitivity and specificity of 96.4 % and 83.8 %, respectively, although there is substantial variability across studies (Llewellyn et al., 2020). The IWGDF/IDSA rates the evidence for advanced imaging in DFO as moderate because of inconsistency, imprecision, and indirectness (Senneville et al., 2023), highlighting the potential value of integrating complementary variables rather than relying on a single test (Llewellyn et al., 2020).

Recently, machine learning (ML) has been increasingly explored to support diagnostic classification and outcome prediction by modeling complex relationships among clinical, laboratory, and imaging variables. In diabetic-foot care, ML applications have incorporated diverse data sources, including medical imaging, biomarkers, and clinical information, to support diagnostic and clinical decision-making (Guan et al., 2024; Misir, 2025). Across DFO studies, however, the predictors and features used have varied according to the data modality and modeling approach.

Clinical decision-making involves integrating symptoms, wound findings, laboratory results, and vascular status and imaging (Kline et al., 2022). Multimodal ML can integrate these complementary data sources, but the value of this approach should be demonstrated by direct comparison with unimodal models in the same population and appropriate validation rather than being assumed due to model complexity (Kline et al., 2022).

Recent reviews of machine learning in diabetic-foot care have described applications involving thermography, ulcer photography, structured clinical data, sensors, risk prediction, and outcome classification, but these reviews were not focused on DFO-specific clinical endpoints and reference standards (Guan et al., 2024; Misir, 2025). Therefore, this systematic review summarizes the evidence regarding ML for DFO diagnosis, risk prediction, and treatment outcomes, focusing on data sources, clinical endpoints, reference standards, model performance, validation methods, and gaps in the evidence.

## 2 Materials and methods

### 2.1 Review design and reporting

This systematic review was conducted and reported in accordance with the Preferred Reporting Items for Systematic Reviews and Meta-Analyses (PRISMA) 2020 statement and the TRIPOD-SRMA guideline for systematic reviews of prediction model studies (Page et al., 2021; Snell et al., 2023). The review questions and extraction framework were informed by the CHARMS checklist (Moons et al., 2014). Relevant reporting considerations from TRIPOD+AI were also used to characterize machine-learning and deep-learning studies (Moons et al., 2025; Collins et al., 2024b). An internal review protocol was prepared before study initiation. The review was not registered, and the protocol is not publicly available; however, it can be provided by the corresponding author upon reasonable request.

### 2.2 Eligibility criteria

We included original human studies evaluating, developing, or interpreting ML or artificial intelligence (AI) models relevant to DFO. Eligible endpoints were DFO detection, differentiation from other diabetic-foot conditions, lesion localization, future DFO development, and clinical outcomes in patients with established DFO.

Diabetic-foot studies that complemented the DFO model by using osteomyelitis as a predictor, severity component, or target of model interpretability were included as these could inform candidate predictors and future DFO models. Demographic, clinical, wound, vascular, laboratory, photographic, radiographic, MRI, and radiomic data were eligible inputs. Studies had to develop, evaluate, or interpret an eligible ML and/or AI model and report a quantitative performance measure. Studies using conventional statistical models only were excluded unless used as comparators. Exclusion criteria were reviews, meta-analyses, editorials, letters, case reports, animal studies, non-peer-reviewed reports without full text, studies published before 2000, and studies without an eligible ML and/or AI model.

### 2.3 Information sources and search strategy

A comprehensive search was conducted using Ovid MEDLINE, Ovid Embase, Scopus, and Web of Science from 1 January 2000 to 15 May 2026 using controlled vocabulary and free-text terms for diabetic-foot/ulcer, osteomyelitis/bone infection, AI/ML, prediction/classification, diagnosis, validation, model performance, and treatment outcomes. Searches were limited to English-language human studies. Full database-specific strategies are provided in the Supplement.

### 2.4 Study selection

Records were imported into Covidence (Veritas Health Innovation, Melbourne, Australia), and duplicates were removed. Two reviewers (FP and SMAA) independently screened titles and/or abstracts and assessed potentially eligible full texts. Disagreements were resolved by discussion, and full-text exclusion reasons were recorded in the PRISMA flow diagram.

### 2.5 Data extraction

A standardized extraction form based on the CHARMS was piloted and refined. Two reviewers (FP and SMAA) independently extracted and checked study characteristics, population, clinical endpoints, intended use, prediction time point, reference standards, predictors, imaging characteristics, preprocessing, feature selection, algorithms, model development and validation methods, discrimination, calibration, classification and localization metrics, and explainability methods.

When studies evaluated multiple algorithms, all eligible models were extracted. For the main tables, we presented the authors' primary or final model; when none was specified, we selected the model with the most complete clinically relevant validation results. Development and internal- and external-validation results were extracted separately.

### 2.6 Model endpoint classification

Synthesis was aggregated by clinical endpoint rather than algorithm type. Studies were categorized into (1) binary DFO detection and/or classification; (2) multiclass or differential diagnosis with DFO; (3) DFO localization or explainability; and (4) complementary diabetic-foot models with osteomyelitis as a predictor, severity component, or model context. This approach allowed for the comparison of models addressing similar clinical questions while avoiding comparisons across heterogeneous model types.

### 2.7 Risk of bias, quality, and applicability

Risks of bias, methodological quality, and applicability were assessed using PROBAST + AI across four domains: participants/data sources, predictors, outcome, and analysis. Two reviewers (FP and SMAA) independently assessed signalling questions and reached consensus; pre-consensus assessments were retained, and inter-rater agreement was measured using percentage agreement and Cohen's kappa. The disagreements and their type were recorded. For explainability-oriented studies, PROBAST + AI was applied to the underlying prediction model, and explainability methods were summarized narratively (Moons et al., 2025).

### 2.8 Data synthesis and statistical analysis

Meta-analysis was not performed because studies differed substantially in terms of populations, endpoints, input modalities, reference standards, algorithms, validation strategies, thresholds, and units of analysis. Findings were synthesized narratively and in structured tables. Performance estimates were reported as presented, with confidence intervals where available, and were not interpreted as evidence of superiority across studies with different endpoints, populations, or validation designs.

## 3 Results

### 3.1 Study selection

The search identified 2343 records (Scopus, 871; Embase, 626; Web of Science, 453; MEDLINE, 393). After removing 989 duplicates, 1354 records underwent title and/or abstract screening, of which 1309 were excluded at this stage. A total of 45 full-text reports were assessed, of which 35 were excluded for not developing an eligible ML model, leaving 10 studies for narrative synthesis (Fig. 1) (Yasin et al., 2025; Abbas et al., 2026; Álvaro-Afonso et al., 2025; Cakir et al., 2024; Cuce et al., 2023; Gao et al., 2025; Gudivaka et al., 2025; Kelly et al., 2024; Nie et al., 2026; Yogapriya et al., 2022).

**Figure 1:**
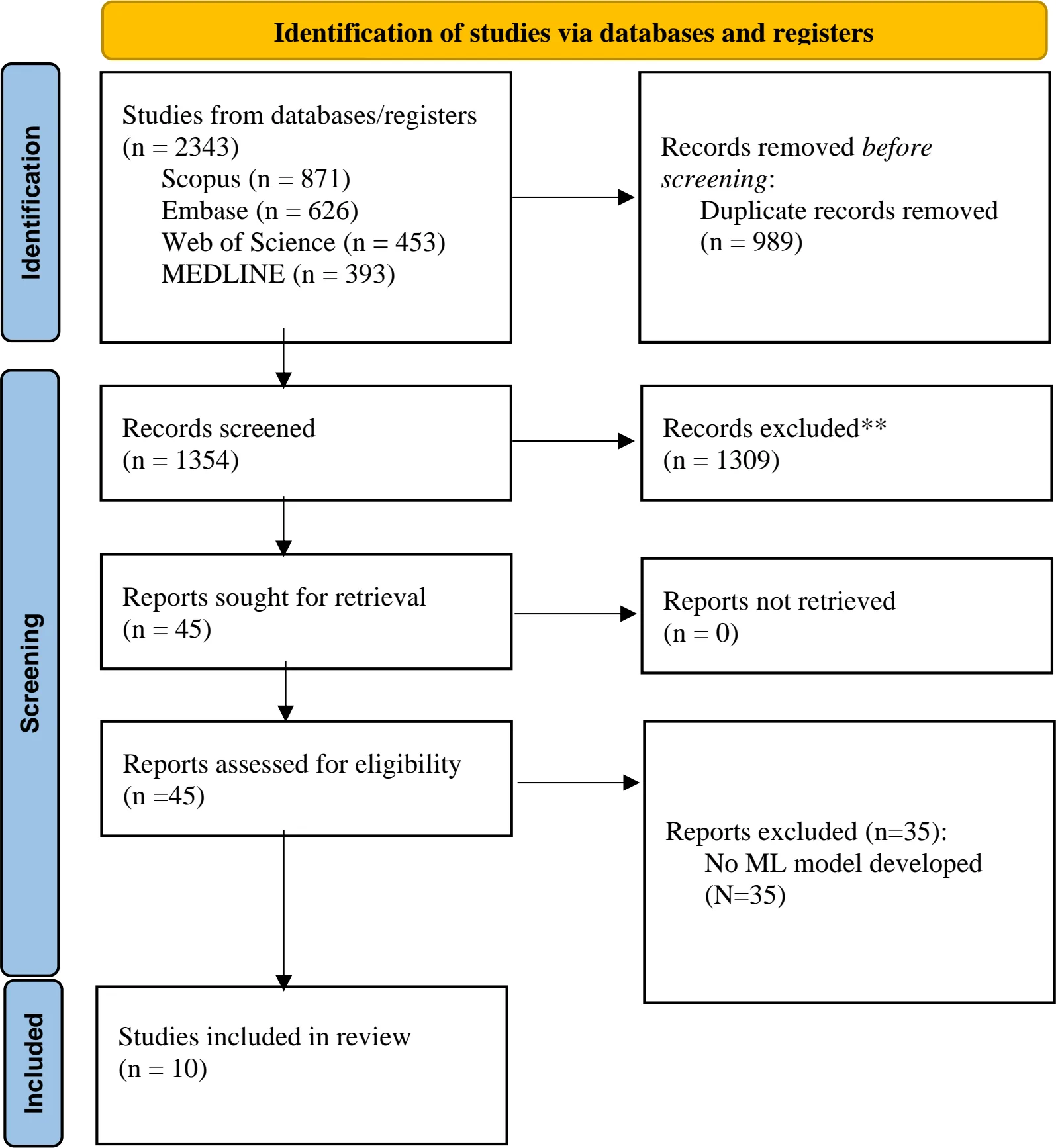
PRISMA flow diagram of study identification, screening, eligibility assessment, and inclusion.

### 3.2 Overall study and model characteristics

The 10 included studies were published between 2022 and 2026 and used data from China (3), Türkiye (2), Spain, Australia (2), USA, and India. Most were retrospective or secondary analyses. Abbas et al. (2026) used radiographs collected prospectively for model development.

Five studies directly addressed DFO diagnosis, while four evaluated complementary diabetic-foot endpoints using osteomyelitis as a predictor, severity component, or model context; also, one additional study evaluated model explainability. Among direct DFO studies, one used clinical/laboratory variables, two plain radiographs, and two MRI inputs. None evaluated incident DFO or DFO-specific treatment outcomes, including treatment response, healing, recurrence, treatment failure, amputation, or mortality. Study characteristics are summarized in Tables 1 and 2.

**Table 1:** Characteristics of studies with a direct diabetic-foot osteomyelitis endpoint. DFO: diabetic-foot osteomyelitis; DFI: diabetic-foot infection; CNO: Charcot neuropathic osteoarthropathy; CV: cross-validation; LR: logistic regression; MLP: multilayer perceptron; NR: not reported; ROI: region of interest; HbA1c: glycated hemoglobin; ESR: erythrocyte sedimentation rate; GLCM: gray-level co-occurrence matrix; GLDM: gray-level dependence matrix; SDHGLE: small-dependence high-gray-level emphasis; MRI: magnetic resonance imaging; GBM: gradient-boosting machine; CNN: convolutional neural network.

Study	Design/setting	Sample used in model and split	Model(s)	Endpoint	Reference standard	Variables/model inputs
Yasin et al. (2025); China	Retrospective, two-center diagnostic study (2015–2024).	N=3612 : DFO 1913; soft-tissue DFI without DFO 1699. Development n=3271 ( ∼2453 train/ ∼818 internal validation); external n=341 .	LightGBM	DFO vs. soft-tissue DFI without bone involvement.	Composite clinical, laboratory, MRI, microbiology/histopathology, clinical course, and treatment response diagnosis.	Age, HbA1c, creatinine, albumin, sodium, ESR.
Cuce et al. (2023); Türkiye	Retrospective, single-center MRI radiomics study (2020–2022).	N=70 patients: DFO 24; Charcot 17; trauma 29. T1 ROIs = 299; T2 ROIs = 301; 10-fold CV.	MLP (best); LR comparator	DFO one-vs.-all; secondary three-class DFO/Charcot/trauma differentiation.	DFO histology; Charcot clinical/laboratory follow-up and response to offloading; trauma controls.	14 selected T1/T2 radiomic features, including GLCM contrast, GLDM SDHGLE, percentiles, skewness, correlation, zone entropy, inverse variance, and sum average.
Cakir et al. (2024); Türkiye	Retrospective, single-center MRI deep-learning study.	N=148 patients: DFO 64; CNO 23; trauma 61. T1 ROIs = 679 (3395 augmented); T2 ROIs = 714 (3570 augmented); five repeated splits with 3-fold CV.	EfficientNet-b0 (best); ResNet-50 comparator	DFO one-vs.-all; secondary three-class DFO/CNO/trauma differentiation.	DFO histology; CNO clinical/laboratory response; trauma controls.	Radiologist-segmented T1/T2 MRI ROIs; learned CNN features (no hand-crafted predictors).
Álvaro-Afonso et al. (2025); Spain	Retrospective diagnostic study in a specialized diabetic-foot unit (2020–2024).	N=168 : DFO 123; no DFO 45. Reported 80:20 split ( ∼134 train/ ∼34 validation).	ResNet-50	Binary DFO detection on plain radiographs.	Surgical bone-biopsy histopathology.	Plain radiographs plus ulcer-location information; learned image features.
Abbas et al. (2026); Australia	Prospectively collected, single-center radiograph dataset; internal model development.	N=225 patients/1227 images. Train 179 patients/981 images; validation 23/123; test 23/123. DFO-positive labels of images 628; negative 599.	DualBack-GFT	Primary image-level DFO classification; secondary lesion localization.	Consensus radiologist annotations and bounding boxes.	Dorsoplantar/oblique radiographs, expert bounding boxes, and learned global/local image features.

**Table 2:** Characteristics of complementary diabetic-foot ulcer-relevant studies. ABI: ankle-brachial index; DFU: diabetic-foot ulcer; DFO: diabetic-foot osteomyelitis; IWGDF/IDSA: International Working Group on the Diabetic Foot/Infectious Diseases Society of America; NR: not reported; XAI: explainable artificial intelligence; EHR: electronic health records; ML: machine learning.

Study	Design/setting	Sample used in model and split	Model	Endpoint	Reference standard	Variables/model inputs
Nie et al. (2026); China	Retrospective, two-center EHR study of hospitalized DFU patients (2020–2024)	N=800 . Development n=500 : train 350/test 150; independent external validation n=300	Random forest	Concurrent wound-infection classification in DFU	IWGDF/IDSA clinical infection criteria; microbiology not mandatory	Age, diabetes duration, ABI, ulcer area, vascular complications, osteomyelitis
Gao et al. (2025); China	Retrospective, single-center hospitalized DFU cohort (2015–2024)	N=599 . Any-amputation model train/test 480/119; minor 440/109; major 354/88	XGBoost	Any, minor, and major in-hospital amputation	Index hospitalization amputation outcome	29 variables across demographics, comorbidities, inflammation, glycemia, renal/nutritional markers, wound severity, osteomyelitis, and vascular intervention
Yogapriya et al. (2022); India	Secondary analysis of an existing DFU photograph dataset	5890 original images augmented to 29 450 images; three train/test splits were evaluated ( 70:30 , 80:20 , and 90:10 ); the final training workflow used 21 204/5890/2356 images for training/validation/testing	DFINET CNN	Infected vs. non-infected DFU photographs	Senior-physician image label; exact clinical/microbiologic standard NR	Color DFU photographs only; no structured clinical variables
Gudivaka et al. (2025); USA	Computational DFU image classification study; dataset and setting unclear	Patient/image total and train/validation/test counts NR	Advanced ML practical method (unclear)	DFU severity/grade classification and infection clustering	Reference standard NR; osteomyelitis/bone-involvement definitions inconsistent	Mixed/unclear photographs, thermograms, or DICOM-derived images; no reliable final feature set
Kelly et al. (2024); Australia	Conference XAI/methods study using DFO radiograph patches	882 balanced patches ( ∼441 DFO + / ∼441 DFO - ); training/validation/test and patient-level counts NR	VGG16 + Mtree-CCEA	Counterfactual explanation quality for a fixed DFO classifier	Professionally labeled radiographs; clinical reference standard NR	224×224 12-bit radiograph patches; learned VGG16 features and counterfactual pixel changes

### 3.3 Models directly evaluating DFO

#### 3.3.1 Binary DFO detection and classification

Three studies evaluated machine-learning models for binary DFO detection or classification (Abbas et al., 2026; Álvaro-Afonso et al., 2025; Yasin et al., 2025) Yasin et al. (2025) developed a two-center model using clinical and laboratory variables to differentiate DFO from soft-tissue diabetic-foot infection (DFI) without bone involvement and reported both internal- and external-validation results (Yasin et al., 2025).

Álvaro-Afonso et al. (2025) evaluated a ResNet-50 model using radiographs with ulcer-location information and histopathological confirmation from surgical bone biopsy (Álvaro-Afonso et al., 2025).

Abbas et al. (2026) developed a deep-learning model for image-level DFO classification using plain radiographs with patient-level training and evaluation splits (Abbas et al., 2026) . Model inputs, reference standards, and performance measures are summarized in Tables 1 and 3.

**Table 3:** Model performance and validation for studies with a direct diabetic-foot osteomyelitis endpoint. DFO: diabetic-foot osteomyelitis; DFI: diabetic-foot infection; AUC: area under the receiver operating characteristic curve; CI: confidence interval; CV: cross-validation; NR: not reported; PPV/NPV: positive/negative predictive value; ROI: region of interest. “Yes” indicates that an internal-validation method was reported and does not by itself imply low risk of bias.

Study	Endpoint	AUC	Sensitivity	Specificity	Other reported performance	Internal validation	External validation
Yasin et al. (2025)	DFO vs. soft-tissue DFI without DFO	0.879 internal; 0.942 external	80.1 %	78.2 %	Accuracy 78.9 %; PPV 69.9 %; NPV 86.2 %	Yes – 75:25 holdout (2453/818) plus nested CV	Yes – independent center, n=341
Cuce et al. (2023)	OM one-vs.-all, T2-weighted MRI, MLP	0.909	85.9 %	89.9 %	Accuracy 88.0 %; PPV 88.4 %; NPV 87.7 %	Yes – 10-fold CV on 299 T1 and 301 T2 ROIs; patient-level grouping NR	No
Cakir et al. (2024)	OM one-vs.-all, T1-weighted MRI, EfficientNet-b0	0.998	97.6 %	98.7 %	NR	Yes – five repeated splits with 3-fold CV on augmented ROIs; fixed counts NR	No
Álvaro-Afonso et al. (2025)	DFO present vs. absent	NR	92.8 %	4.4 %	Accuracy 69.1 %; PPV 72.6 %; NPV 18.2 %; $F_{1}$ score 81.4 %	Yes – reported 80:20 split (134/34)	No
Abbas et al. (2026)	Image-level DFO classification	0.968 (95 % CI 0.930–1.000)	90.5 %	95.0 %	Balanced accuracy 92.7 %; $F_{1}$ score 92.7 %	Yes – patient-level train/validation/test: 179/23/23 patients	No

#### 3.3.2 Multiclass and differential diagnosis of DFO

Two studies evaluated MRI-based ML models for DFO classification and differential diagnosis (Cakir et al., 2024; Cuce et al., 2023). Cuce et al. developed a radiomics model using segmented T1- and T2-weighted MRI regions and a multilayer perceptron with cross-validation (Cuce et al., 2023). Cakir et al. (2024) evaluated deep-learning models based on segmented MRI regions using transfer-learning architectures for DFO classification and differential diagnosis with Charcot neuroarthropathy and trauma (Cakir et al., 2024). MRI dataset characteristics, model inputs, validation approaches, and performance measures are summarized in Tables 1 and 3.

#### 3.3.3 DFO localization and model explainability

In addition to image-level classification, Abbas et al. (2026) evaluated patch-based lesion localization and reported recall of 82.4 %, precision of 42.2 %, and an $F_{1}$ score of 55.8 % (Abbas et al., 2026).

Kelly et al. (2024) evaluated an explainability approach on a previously developed deep-learning classifier for DFO detection from radiograph patches. The study concentrated on visual explanations and not diagnostic performance; although classifier accuracy was reported, AUC (area under the receiver operating characteristic curve), sensitivity, specificity, patient-level data separation, and external validation were not (Kelly et al., 2024).

### 3.4 Complementary DFO-relevant machine-learning studies

#### 3.4.1 Osteomyelitis used as a model predictor

Nie et al. (2026) developed a random-forest model for wound infection classification in 800 hospitalized patients with DFU, with six final predictors including osteomyelitis. The AUCs were 0.853 in the internal test cohort and 0.832 in an independent external cohort (Nie et al., 2026).

Gao et al. (2025) developed XGBoost models for non-amputation, minor amputation, and major amputation outcomes among 599 hospitalized patients with DFU, with osteomyelitis included among 29 candidate variables. The AUCs were 0.772 for non-amputation, 0.800 for minor amputation, and 0.977 for major amputation (Gao et al., 2025).

#### 3.4.2 Related infection and severity classification

Yogapriya et al. (2022) designed a convolutional neural network to classify infected versus non-infected DFUs using ulcer photographs. There was no separate assessment of osteomyelitis and no DFO-specific performance reported (Yogapriya et al., 2022).

Gudivaka et al. evaluated an ML framework for DFU severity classification, incorporating osteomyelitis or bone involvement into severity definitions. DFO was not assessed as an independent endpoint, and details on the dataset, reference standard, validation strategy, and DFO-specific performance were unavailable (Gudivaka et al., 2025).

### 3.5 Model inputs and predictor selection approaches

Direct DFO models utilized structured clinical and/or laboratory or imaging data; none combined clinical or laboratory variables with radiographic or MRI features. Reported predictors were demographic, glycemic, renal, inflammatory, and nutritional variables. Complementary diabetic-foot models included vascular, wound, inflammatory, and osteomyelitis-related variables. Predictors were selected by different methodologies across the studies, including expert and statistical methods, tree-based importance, explainability, radiomic selection, and learned deep-learning features. Tables 1 and 2 summarize detailed predictors and modeling approaches.

**Table 4:** Model performance and validation for complementary DFO-relevant studies. AUC: area under the receiver operating characteristic curve; CV: cross-validation; DFU: diabetic-foot ulcer; DFO: diabetic-foot osteomyelitis; MCC: Matthews correlation coefficient; NR: not reported. ^*^ For Gao et al. (2025), performance metrics are presented as reported by the authors for the any-, minor-, and major-amputation models, respectively.

Study	Endpoint	AUC	Sensitivity	Specificity	Other reported performance	Internal validation	External validation
Nie et al. (2026)	Wound infection in DFU	0.853 internal; 0.832 external	NR	NR	Internal CV AUC 0.820; external-cohort CV AUC 0.744	Yes – train/test 350/150 plus cross-validation	Yes – independent center, n=300
Gao et al. (2025)	Non-amputation/minor amputation/major amputation^*^	0.772, 0.800, 0.977	75.7 %, 78.1 %, 98.7 %	77.8 %, 69.4 %, 88.9 %	NPV 66.0 %, 61.0 %, 88.9 %; PPV 84.8 %, 83.8 %, 98.7 %	Yes – 80:20 holdout plus 5-fold CV; test n=119/109/88	No
Yogapriya et al. (2022)	Infected vs. non-infected DFU images	NR	90.57 %	93.49 %	Accuracy 91.98 %; precision 93.72 %; $F_{1}$ score 92.12 %; MCC 0.84	Yes – image-level 90:10 train/test split after augmentation; 27 094/2356	No
Gudivaka et al. (2025)	DFU grade/severity classification	NR	NR	NR	Maximum grade-specific classification accuracy 93.75 %	Unclear – validation procedure not reproducibly reported	No
Kelly et al. (2024)	Counterfactual explanation of DFO classifier	NR	NR	NR	Underlying classifier accuracy 78.5 %	Unclear – classifier split and patient partition	No

### 3.6 Model validation and predictive performance

All five direct DFO studies reported internal validation using holdout; cross-validation; repeated data splitting; or separate training, validation, and testing groups. Only Yasin et al. (2025) performed independent external validation of a direct DFO model (Yasin et al., 2025). Among complementary studies, Nie et al. (2026) also externally validated its wound infection model in an independent hospital cohort (Nie et al., 2026).

AUCs among direct DFO models ranged from 0.879 to 0.998 but were not directly comparable because studies differed in terms of populations, endpoints, reference standards, modalities, case mix, units of analysis, and validation designs. Calibration was reported inconsistently, and several studies failed to report confidence intervals, clinically defined thresholds, or full sensitivity and specificity. Tables 3 and 4 summarize the performance and validation.

### 3.7 Risk of bias, quality, and applicability

The quality assessment is summarized in Table S1 in the Supplement. Most studies were judged at high overall risk of bias (applicability concerns were present in some studies), and methodological quality was variable. Inter-reviewer agreement was moderate to almost perfect for PROBAST + AI domains. Cohen's κ showed a substantial agreement for participants ( κ=0.65 ), a moderate agreement for predictors ( κ=0.54 ) and outcome ( κ=0.55 ), and an almost perfect agreement for overall applicability ( κ=1.00 ). Similar analysis and overall risk of bias ratings were seen across studies ( κ=0.00 due to limited rating variability; kappa paradox). All assessments were discussed, and a consensus was reached for use in the final analysis.

## 4 Discussion

In this systematic review of ML applications in DFO, we identified 10 studies evaluating models for direct DFO diagnosis, related diabetic-foot outcomes, or model interpretation. The available evidence was primarily focused on diagnostic applications, with direct DFO models using either structured clinical and laboratory variables or imaging-based inputs, including plain radiographs and MRI. Only one of the five direct DFO diagnostic studies reported independent external validation, and no included study developed a fully multimodal model integrating clinical or laboratory information with radiographic or MRI features. Overall, the current evidence does not establish a sufficiently consistent basis for comparable or generalizable ML approaches to DFO given substantial heterogeneity in clinical endpoints, reference standards, input modalities, validation strategies, and reporting practices.

Although several included studies reported high discrimination, direct comparison of AUC, sensitivity, specificity, or other performance measures across models are not appropriate.

Among direct DFO diagnostic models, clinical and laboratory-based approaches demonstrated the potential value of routinely available patient-level information. These findings indicate that routinely collected clinical data can be useful for the classification of DFO, but the generalizability of such models relies on consistent definitions of outcomes, proper selection of predictors, and validation in different clinical settings. Prediction models may demonstrate strong discrimination when developed in specific populations but may lose performance when applied to patients with different case mix characteristics or clinical workflows (Collins et al., 2024a; Riley et al., 2024).

Interpretation of imaging-based models must consider the reference standard, the structure of the dataset, and the validation approach. Plain radiographs have limited sensitivity for early DFO and may show findings that overlap with other conditions affecting the diabetic foot. Therefore, careful selection of reference standards is essential when evaluating image-based models (Dinh et al., 2008; Senneville et al., 2008). Similarly, MRI-based models evaluated by Cuce et al. (2023) and Cakir et al. (2024) reported high internal discrimination for differentiating DFO from other bone-related conditions; however, these studies included relatively small cohorts and used segmented or augmented image data, limiting confidence in the generalizability of their performance estimates (Cakir et al., 2024; Cuce et al., 2023; Varoquaux and Cheplygina, 2022).

One major limitation was the lack of external validation. Cross-validation, holdout testing, and repeated data splitting evaluate performance on the available data but do not determine accuracy across institutions, populations, or clinical settings (Collins et al., 2024a). External validation is essential because changes in disease prevalence, patient characteristics, imaging protocols, laboratory measurements, and clinical decision-making pathways may significantly affect model performance (Riley et al., 2024). Furthermore, discrimination by itself does not establish clinical utility. Prediction models for clinical decision-making should also show reliable calibration, clinically meaningful thresholds, and evidence of clinical utility beyond statistical performance measures (Collins et al., 2024a; Efthimiou et al., 2024). In the present review, calibration reporting was inconsistent, and decision-analytic approaches were rarely evaluated, limiting the assessment of potential clinical impact.

The complementary diabetic-foot studies included in this review provided additional information regarding potential predictors and modeling approaches but should not be interpreted as DFO diagnostic models.

Current clinical assessment of DFO already relies on multiple complementary data sources, including wound characteristics, probe-to-bone findings, inflammatory markers, vascular status, plain radiography, and MRI when needed (Senneville et al., 2023). However, previous ML studies have generally evaluated these information sources separately: clinical and laboratory variables were used without imaging, while radiograph and MRI models were developed without broader patient-level information (Abbas et al., 2026; Álvaro-Afonso et al., 2025; Yasin et al., 2025; Cakir et al., 2024; Cuce et al., 2023). Future research should therefore investigate whether combining clinical and imaging data provides incremental value compared with unimodal approaches. Multimodal models should be developed and evaluated using the same patient population, standardized reference standards, and independent validation cohorts, allowing assessment of whether integration improves discrimination, calibration, and clinical utility rather than assuming benefit from increased model complexity alone (Collins et al., 2024a; Kline et al., 2022).

Future studies should focus on clinically available predictors, excluding variables collected after the intended prediction time or variables that directly contribute to the diagnostic reference standard and ensuring proper patient-level separation during imaging preprocessing and model development (Efthimiou et al., 2024; Varoquaux and Cheplygina, 2022). A clinically meaningful multimodal DFO model should be compared against clinical-only and imaging-only models, include external validation, report calibration and decision-analytic measures, and evaluate performance across relevant patient subgroups before implementation in clinical practice (Collins et al., 2024a, b; Riley et al., 2024).

This systematic review has several strengths. We performed a comprehensive search across multiple databases, used a structured extraction framework informed by CHARMS, separated direct DFO models from complementary diabetic-foot models, and synthesized evidence according to clinical endpoint rather than algorithm type. This approach reduced the risk of misusing general diabetic-foot-infection or severity models as DFO diagnostic models and offered a summary of model inputs, validation strategies, and reporting approaches. This review also has several limitations. There was considerable heterogeneity in populations, endpoints, reference standards, input modalities, and validation methods, which hampered quantitative synthesis and direct comparison of model performance. Furthermore, most studies had limited external validation and incomplete reporting of calibration, preprocessing procedures, and model development details, limiting the assessment of reproducibility and clinical applicability.

## 5 Conclusions

Most of the ML research in DFO has focused on diagnostic classification using either structured clinical and laboratory variables or imaging-based approaches. However, the current evidence does not establish a sufficiently consistent or generalizable ML approach to DFO given substantial heterogeneity in study design, clinical endpoints, reference standards, input modalities, validation strategies, and reporting. None of the included studies developed a multimodal model that incorporated clinical or laboratory data with radiographic or MRI features, and none of the studies directly assessed prediction of incident DFO or DFO-specific treatment outcomes. Future research should establish standardized clinical endpoints and reference standards and focus on the development and validation of multimodal models integrating complementary information from clinical and imaging sources, testing clinical-only, imaging-only and combined approaches on the same cohorts, with external validation, calibration assessment, and clinical utility analysis prior to implementation in practice.

## Supplement

10.5194/jbji-11-623-2026-supplement The supplement related to this article is available online at https://doi.org/10.5194/jbji-11-623-2026-supplement.

## Data Availability

All data used in this study were obtained from previously published articles cited in the paper. The extracted dataset supporting the findings of this study is available from the corresponding author upon reasonable request.
