# Supplementary material for: Machine learning in diabetic-foot osteomyelitis: current applications, challenges, and future directions
Source: J Bone Jt Infect. 2026 Oct 13;11(5):623–34. doi: 10.5194/jbji-11-623-2026 (PMC13662808; doi:10.5194/jbji-11-623-2026)
Supplement: Supplementary file 1 [file jbji-11-623-2026-supplement.pdf]

Supplement of J. Bone Joint Infect., 11, 623–634, 2026
https://doi.org/10.5194/jbji-11-623-2026-supplement
© Author(s) 2026. CC BY 4.0 License.

Supplement of

Machine learning in diabetic-foot osteomyelitis: current applications, challenges, and future directions

Farzad Pourghazi et al.

Correspondence to: Elie F. Berbari (berbari.elie@mayo.edu)

The copyright of individual parts of the supplement might differ from the article licence.

Contents

Search Strategy 1

Supplementary Table 1. 10

Search Strategy

<u>MEDLINE</u>

Ovid MEDLINE(R) and In-Process, In-Data-Review & Other Non-Indexed Citations <1946 to May 15, 2026>

1 exp Diabetic Foot/ 13943

2 exp Foot Ulcer/ 15421

3 exp Diabetes Mellitus/ 568827

4 (diabet* adj3 (foot or feet or pedal or plantar or ulcer* or wound* or infection*)).ti,ab. 28604

5 ("diabetic foot" or "diabetic feet" or "diabetic foot ulcer*" or "diabetes-related foot" or "diabetes related foot" or DFU or DFI).ti,ab. 17365

6 2 and 3 14219

7 1 or 4 or 5 or 6 34136

8 exp Osteomyelitis/ 25922

9 (osteomyelitis or osteomyelitides or "bone infection*" or "bone involvement" or "pedal osteomyelitis" or "foot osteomyelitis" or DFO).ti,ab. 36205

10 8 or 9 44729

11 exp Artificial Intelligence/ 280893

12 exp Machine Learning/ 121788

13 exp Deep Learning/ 41557

14 exp Neural Networks, Computer/ 101122

15 exp Algorithms/ 564708

16 exp Logistic Models/ 160775

17 exp Prognosis/ 2162196

18 exp "Sensitivity and Specificity"/ 713664

19 exp ROC Curve/ 86622

20 (artificial intelligence or AI or "machine learning" or ML or "deep learning" or "neural network*" or "convolutional neural network*" or CNN or ResNet or XGBoost or "random forest*" or "support vector machine*" or SVM or "decision tree*" or "gradient boosting" or classifier* or classification or algorithm* or "computer-aided" or "computer assisted" or automated).ti,ab. 2391471

21 ((predict* or prognos* or diagnos* or detect* or classif* or discriminat* or differentiat* or stratificat*) adj3 (model* or score* or tool* or algorithm* or classifier* or rule* or system* or nomogram*)).ti,ab. 826299

22 ("prediction model*" or "predictive model*" or "diagnostic model*" or "prognostic model*" or "risk model*" or "risk score*" or nomogram* or "clinical prediction rule*" or

"clinical prediction model*" or "multivariable model*" or "multivariate model*" or "logistic regression model*" or "Cox regression model*").ti,ab. 374274

23 (AUC or AUROC or "area under the curve" or ROC or "receiver operating characteristic" or calibration or discrimination or "external validation" or "internal validation" or "cross-validation" or "cross validation" or sensitivity or specificity or accuracy or "F1 score" or precision or recall).ti,ab. 2674233

24 ((treatment or antibiotic* or surgical or surgery or amputation or remission or recurrence or reinfection or relapse or healing or failure or outcome*) adj3 (predict* or prognos* or model* or score* or classifier* or algorithm*)).ti,ab. 464614

25 11 or 12 or 13 or 14 or 15 or 16 or 17 or 18 or 19 or 20 or 21 or 22 or 23 or 24 7405853

26 7 and 10 and 25 757

27 limit 26 to english language 720

28 limit 27 to yr="2000 -Current" 647

29 limit 28 to humans 561

30 exp animals/ not humans/ 5450259

31 case reports.pt. 2549107

32 review.pt. 3455559

33 systematic review.pt. 327356

34 meta-analysis.pt. 226407

35 editorial.pt. 752809

36 letter.pt. 1327597

37 30 or 31 or 32 or 33 or 34 or 35 or 3613236375

38 29 not 37 393

<u>Embase <1988 to 2026 Week 20></u>

1 exp diabetic foot/ 27171

2 exp foot ulcer/ 10278

3 exp diabetes mellitus/ 1463621

4 (diabet* adj3 (foot or feet or pedal or plantar or ulcer* or wound* or infection*)).ti,ab. 40218

5 ("diabetic foot" or "diabetic feet" or "diabetic foot ulcer*" or "diabetes-related foot" or "diabetes related foot" or DFU or DFI).ti,ab. 25492

6 2 and 37680

7 1 or 4 or 5 or 652073

8 exp osteomyelitis/ 46625

9 (osteomyelitis or osteomyelitides or "bone infection*" or "bone involvement" or "pedal osteomyelitis" or "foot osteomyelitis" or DFO).ti,ab. 40858

10 8 or 9 62574

11 exp artificial intelligence/ 195002

12 exp machine learning/ 718939

13 exp machine learning/ 718939

14 exp deep learning/ 108170

15 exp artificial neural network/ 163804

16 exp algorithm/ 829701

17 exp logistic regression/ 180290

18 exp prognosis/ 1011165

19 exp receiver operating characteristic/ 321018

20 (sensitivity or specificity).ti,ab. 1783476

21 (artificial intelligence or AI or machine learning or ML or deep learning or neural network* or convolutional neural network* or CNN or ResNet or XGBoost or random forest* or support vector machine* or SVM or decision tree* or gradient boosting or classifier* or classification or algorithm* or computer-aided or computer assisted or automated).ti,ab. 3225411

22 ((predict* or prognos* or diagnos* or detect* or classif* or discriminat* or differentiat* or stratificat*) adj3 (model* or score* or tool* or algorithm* or classifier* or rule* or system* or nomogram*)).ti,ab. 1100018

23 (prediction model* or predictive model* or diagnostic model* or prognostic model* or risk model* or risk score* or nomogram* or clinical prediction rule* or clinical prediction model* or multivariable model* or multivariate model* or logistic regression model* or Cox regression model*).ti,ab. 539880

24 (AUC or AUROC or area under the curve or ROC or receiver operating characteristic or calibration or discrimination or external validation or internal validation or cross-validation or cross validation or sensitivity or specificity or accuracy or F1 score or precision or recall).ti,ab. 3254277

25 ((treatment or antibiotic* or surgical or surgery or amputation or remission or recurrence or reinfection or relapse or healing or failure or outcome*) adj3 (predict* or prognos* or model* or score* or classifier* or algorithm*)).ti,ab. 712711

26 ((treatment or antibiotic* or surgical or surgery or amputation or remission or recurrence or reinfection or relapse or healing or failure or outcome*) adj3 (predict* or prognos* or model* or score* or classifier* or algorithm*)).ti,ab. 712711

27 11 or 12 or 13 or 14 or 15 or 16 or 17 or 18 or 19 or 20 or 21 or 22 or 23 or 24 7691045

28 7 and 10 and 27 880

29 limit 28 to english language 835

30 limit 29 to yr="2000 -Current" 785

31 limit 30 to human 764

32 review.pt. 3412612

33 editorial.pt. 841703

34 letter.pt. 1289675

35 (case report or case reports).ti. 467745

36 32 or 33 or 34 or 35 5975769

37 31 not 36 626

<u>Scopus 871</u>

TITLE-ABS-KEY (("diabetic foot" OR "diabetic feet" OR "diabetic foot ulcer*" OR "diabetes-related foot" OR "diabetes related foot" OR DFU OR DFI OR ((diabet* OR "diabetes mellitus") W/3 (foot OR feet OR pedal OR plantar OR ulcer* OR wound* OR infection*))) AND (osteomyelitis OR osteomyelitides OR "bone infection*" OR "bone involvement" OR "pedal osteomyelitis" OR "foot osteomyelitis" OR DFO) AND ("artificial intelligence" OR AI OR "machine learning" OR ML OR "deep learning" OR "neural network*" OR "convolutional neural network*" OR CNN OR ResNet OR XGBoost OR "random forest*" OR "support vector machine*" OR SVM OR "decision tree*" OR "gradient boosting" OR classifier* OR classification OR algorithm* OR "computer-aided" OR "computer assisted" OR automated OR ((predict* OR prognos* OR diagnos* OR detect* OR classif* OR discriminat* OR differentiat* OR stratificat*) W/3 (model* OR score* OR tool* OR algorithm* OR classifier* OR rule* OR system* OR nomogram*)) OR "prediction model*" OR "predictive model*" OR "diagnostic model*" OR "prognostic model*" OR "risk model*" OR "risk score*" OR nomogram* OR "clinical prediction rule*" OR "clinical prediction model*" OR "multivariable model*" OR "multivariate model*" OR "logistic regression model*" OR "Cox regression model*" OR AUC OR AUROC OR "area under the curve" OR ROC OR "receiver operating characteristic" OR

calibration OR discrimination OR "external validation" OR "internal validation" OR "cross-validation" OR "cross validation" OR sensitivity OR specificity OR accuracy OR "F1 score" OR precision OR recall OR ((treatment OR antibiotic* OR surgical OR surgery OR amputation OR remission OR recurrence OR reinfection OR relapse OR healing OR failure OR outcome*) W/3 (predict* OR prognos* OR model* OR score* OR classifier* OR algorithm*))))) AND PUBYEAR > 1999 AND LANGUAGE (english) AND NOT DOCTYPE (re) AND NOT DOCTYPE (ch) AND NOT DOCTYPE (ed) AND NOT DOCTYPE (le) AND NOT DOCTYPE (no)

Web of Science : 453

TS=(

(

"diabetic foot" OR "diabetic feet" OR "diabetic foot ulcer*" OR "diabetes-related foot" OR "diabetes related foot" OR DFU OR DFI

OR

((diabet* OR "diabetes mellitus") NEAR/3 (foot OR feet OR pedal OR plantar OR ulcer* OR wound* OR infection*))

)

AND

(

osteomyelitis OR osteomyelitides OR "bone infection*" OR "bone involvement" OR "pedal osteomyelitis" OR "foot osteomyelitis" OR DFO

)

AND

(

"artificial intelligence" OR AI OR "machine learning" OR ML OR "deep learning" OR "neural network*" OR "convolutional neural network*" OR CNN OR ResNet OR XGBoost OR "random forest*" OR "support vector machine*" OR SVM OR "decision tree*" OR "gradient boosting" OR classifier* OR classification OR algorithm* OR "computer-aided" OR "computer assisted" OR automated

OR

((predict* OR prognos* OR diagnos* OR detect* OR classif* OR discriminat* OR differentiat* OR stratificat*) NEAR/3 (model* OR score* OR tool* OR algorithm* OR classifier* OR rule* OR system* OR nomogram*))

OR

"prediction model*" OR "predictive model*" OR "diagnostic model*" OR "prognostic model*" OR "risk model*" OR "risk score*" OR nomogram* OR "clinical prediction rule*" OR "clinical prediction model*" OR "multivariable model*" OR "multivariate model*" OR "logistic regression model*" OR "Cox regression model*"

OR

AUC OR AUROC OR "area under the curve" OR ROC OR "receiver operating characteristic" OR calibration OR discrimination OR "external validation" OR "internal validation" OR "cross-

validation" OR "cross validation" OR sensitivity OR specificity OR accuracy OR "F1 score" OR precision OR recall

OR

((treatment OR antibiotic* OR surgical OR surgery OR amputation OR remission OR recurrence OR reinfection OR relapse OR healing OR failure OR outcome*) NEAR/3 (predict* OR prognos* OR model* OR score* OR classifier* OR algorithm*))

)

)

AND LA=(English)

AND PY=(2000-2026)

NOT DT=(Review OR Editorial Material OR Letter OR Meeting Abstract OR Book Chapter)

Table S1. Independent PROBAST+AI assessments of risk of bias and applicability for the included studies. Ratings represent the independent pre-consensus

assessments of the two reviewers. PROBAST+AI, Prediction Model Risk of Bias Assessment Tool–Artificial Intelligence.

**Study**	**Reviewer**	**Participants**	**Predictors**	**Outcome**	**Analysis**	**Overall risk of bias**	**Applicability concern**
**Yasin 2025**	First	Low	Low/Some concerns	Some concerns	High	High	Low/Some concerns
	Second	Low/Some concerns	Low	Low	Some concerns	Some concerns	Low/Some concerns
**Abbas 2026**	First	Some concerns	Low	Some concerns	High	High	Some concerns
	Second	Some concerns	Low	Some concerns	High	High	Some concerns
**Álvaro-Afonso 2025**	First	High	Low	Low	High	High	Some concerns
	Second	High	Low	Low	High	High	Some concerns
**Cakir 2024**	First	High	Low	Some concerns	High	High	Some concerns

	Second	High	Low	Some concerns	High	High	Some concerns
**Cuce 2023**	First	High	Low	Some concerns	High	High	Some concerns
	Second	High	Low	Some concerns	High	High	Some concerns
**Gao 2025**	First	Some concerns	High	High	High	High	High
	Second	Some concerns	Some concerns	Low	High	High	High
**Nie 2026**	First	Low/Some concerns	High	High	High	High	High
	Second	Some concerns	Some concerns	Some concerns	High	High	High
**Gudivaka 2025**	First	High	High	High	High	High	High
	Second	High	High	High	High	High	High
**Kelly 2024**	First	High	High	High	High	High	High
	Second	High	High	High	High	High	High
**Yogapriya 2022**	First	High	High	High	High	High	High
	Second	High	High	High	High	High	High
